# 2-[(*E*)-(1,10-Phenanthrolin-5-yl)imino­meth­yl]phenol methanol monosolvate

**DOI:** 10.1107/S1600536812011890

**Published:** 2012-03-24

**Authors:** Sema Öztürk Yíldírím, Nebahat Demirhan, Fikriye Elmalí, Ray J. Butcher

**Affiliations:** a Inorganic Chemistry Department, Howard University, Washington, DC 20059, USA; b Department of Physics, Faculty of Sciences, Erciyes University, 38039 Kayseri, Turkey; c Yíldíz Technical University, Faculty of Arts and Sciences, Chemistry Department, 34210 Esenler, Istanbul, Turkey; d Department of Chemistry, Howard University, 525 College Street NW, Washington, DC 20059, USA

## Abstract

In the title multi-donor Schiff base compound, C_19_H_13_N_3_O·CH_3_OH, the dihedral angle between the mean planes of the phenanthroline and phenol rings is 59.3 (1)°. The Schiff base mol­ecule is linked to the solvent mol­ecule by an O—H⋯O hydrogen bond. In the crystal, the components are linked by O—H⋯N hydrogen bonds, weak O—H⋯N inter­actions and π–π stacking inter­actions [centroid–centroid distances = 3.701 (1) and 3.656 (1) Å].

## Related literature

For the role played by 1,10-phenanthroline and its derivatives as mol­ecular scaffolds for supra­molecular assemblies, see: Balzani *et al.* (1996 ▶). For the metal-chelating properties of the 1,10-phenanthroline ligand, see: Sammes & Yahioglu (1994 ▶). For the photochemical and redox properties of phenanthroline rings, see: Camren *et al.* (1996 ▶); Bolger *et al.* (1996 ▶); Msood & Hodgson (1993 ▶). For Schiff bases as oxygen-carriers and as photochromic or thermochromic materials, see: Hobday & Smith (1973 ▶); Gul *et al.* (1986 ▶); Can & Bekaroglu (1988 ▶); Avciata *et al.* (1995 ▶, 1998 ▶); Demirhan *et al.* (2002 ▶). For the synthesis of 5-amino-1,10-phenanthroline, see: Gillard & Hill (1974 ▶). For related structures, see: Wu *et al.* (2011 ▶); Fun *et al.* (2010 ▶). For standard bond lengths, see: Allen *et al.* (1987 ▶). 
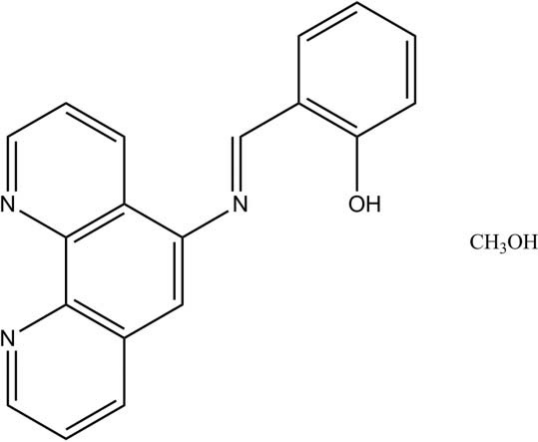


## Experimental

### 

#### Crystal data

C_19_H_13_N_3_O·CH_4_O
*M* *_r_* = 331.37
Monoclinic, 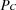

*a* = 11.9398 (12) Å
*b* = 4.6680 (5) Å
*c* = 14.7818 (18) Å
β = 101.961 (11)°
*V* = 805.98 (16) Å^3^
*Z* = 2
Cu *K*α radiation
μ = 0.73 mm^−1^
*T* = 123 K
1.15 × 0.84 × 0.06 mm

#### Data collection

Oxford Diffraction Gemini-R diffractometer
Absorption correction: analytical [*CrysAlis RED* (Oxford Diffraction, 2007 ▶), using a multi-faceted crystal model (Clark & Reid, 1995 ▶)] *T*_min_ = 0.505, *T*_max_ = 0.954
3176 measured reflections
1960 independent reflections
1885 reflections with *I* > 2σ(*I*)
*R*_int_ = 0.030

#### Refinement

*R*[*F* ^2^ > 2σ(*F* ^2^)] = 0.039
*wR*(*F* ^2^) = 0.110
*S* = 1.04
1960 reflections
229 parameters
2 restraints
H-atom parameters constrained
Δρ_max_ = 0.24 e Å^−3^
Δρ_min_ = −0.17 e Å^−3^

### 

Data collection: *CrysAlis PRO* (Oxford Diffraction, 2007 ▶); cell refinement: *CrysAlis PRO*; data reduction: *CrysAlis RED* (Oxford Diffraction, 2007 ▶); program(s) used to solve structure: *SHELXS97* (Sheldrick, 2008 ▶); program(s) used to refine structure: *SHELXL97* (Sheldrick, 2008 ▶); molecular graphics: *SHELXTL* (Sheldrick, 2008 ▶); software used to prepare material for publication: *SHELXTL*.

## Supplementary Material

Crystal structure: contains datablock(s) I, global. DOI: 10.1107/S1600536812011890/jj2127sup1.cif

Structure factors: contains datablock(s) I. DOI: 10.1107/S1600536812011890/jj2127Isup2.hkl

Supplementary material file. DOI: 10.1107/S1600536812011890/jj2127Isup3.cml

Additional supplementary materials:  crystallographic information; 3D view; checkCIF report

## Figures and Tables

**Table 1:** Hydrogen-bond geometry (Å, °)

*D*—H⋯*A*	*D*—H	H⋯*A*	*D*⋯*A*	*D*—H⋯*A*
O1—H1⋯O1*S*	0.84	1.81	2.640 (3)	172
O1*S*—H1*S*⋯N1^i^	0.84	2.01	2.829 (3)	163
O1*S*—H1*S*⋯N2^i^	0.84	2.68	3.242 (3)	126

Symmetry code: (i) 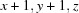.

**Fig. 1.**
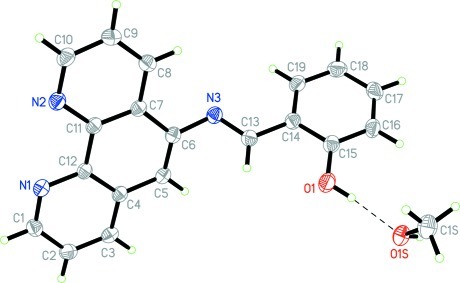
View of the molecular structure of the title compound with the atom-numbering scheme. Displacement ellipsoids are drawn at the 50% probability level for non-hydrogen atoms. Hydrogen bonds are drawn as dashed lines.

**Fig. 2.**
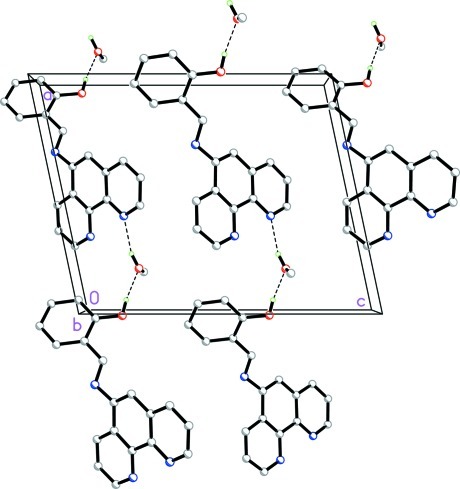
The molecular packing of the title compound. Hydrogen bonds are drawn as dashed lines.

## supplementary crystallographic information

### Comment

1,10-Phenanthroline and its derivatives play important roles as molecular scaffolding for supramolecular assemblies (Balzani *et al.*, 1996). These have played a major role in the development of polypyridyl metal complexes. The metal chelating property of the 1,10-phenanthroline ligand and its derivatives have been utilized in a range of analytical reagents as well as for the development of bioinorganic probes (Sammes & Yahioglu, 1994). The photochemical and redox properties of complexes can be varied systematically through appropriate substitution on the phenanthroline rings (Camren *et al.*, 1996: Bolger *et al.*, 1996: Msood & Hodgson, 1993).

The coordination chemistry of Schiff bases derived from salicylaldehyde has been the subject of many studies because of their interesting properties; *e.g.* as oxygen-carriers to mimic some complicated biological systems, as photochromic or thermochromic materials (Hobday & Smith, 1973: Gul *et al.*, 1986: Can & Bekaroglu, 1988: Avciata *et al.* 1995; Avciata *et al.* 1998; Demirhan *et al.* 2002).

We report here the synthesis and characterization a new multidonor Schiff base derivative, (I), carrying N_3_O donor atoms and prepared from the condensation reaction of 5-amino-1,10-phenanthroline with salicylaldehyde.

The title molecule C_19_H_13_N_3_O.CH_3_OH, crystallized as a methanol monosolvate (Fig. 1). All bond lengths are as expected (Allen *et al.*, 1987) and are comparable to those observed in related structures (Wu *et al.*, 2011; Fun *et al.*, 2010). The molecule is not planar, forming a dihedral angle of 59.3 (1)° between the mean planes of the phenanthroline (N1/N2/C1—C12) and phenol (C14—C19) rings.

In the crystal, O—H···N hydrogen bonds and weak O—H···N intermolecular interactions are observed (Table 1) as well as weak π-π stacking interactions [*Cg*1···*Cg*2 (*x*, 1+*y*, *z*) = 3.701 (1) Å and *Cg*1···*Cg*3 (*x*, 1+*y*,*z*) = 3.656 (1) Å, where *Cg*1(N1/C1—C4/C12), *Cg*2(N2/C7—C11) and *Cg*3(C4—C7/C11—C12) are the centroids of the phenonthroline ring], (Fig. 2).

### Experimental

5-Amino-1,10-phenanthroline (Gillard & Hill, 1974) (1.5 g, 7.69 mmol) in 50 ml absolute methanol was added to salicylaldehyde (0.93 g, 7.69 mmol) dissolved in 20 ml diethylether and 100 ml absolute ethanol. After refluxing this mixture for 4.5 h, the precipitate was filtered off and then washed with water and ether. The product was obtained as a yellow precipitate (70° yield). It was soluble in methanol, ethanol and chloroform. Yield 1.79 g (78%). m.p. 451–453 K; Anal. Calcd. for C_19_H_13_N_3_O.CH_3_OH (299.32 g/mol) C, 74.24; H, 4.38; N, 14.04. Found: C, 74.86; H, 4.12; N, 14.66.

### Refinement

H atoms were placed in geometrically idealized positions and constrained to ride on their parent atoms with O—H = 0.84 Å, C—H = 0.95–0.98 Å and *U*_iso_(H) = 1.2*U*_eq_(C) or *U*_iso_(H) = 1.5 *U*_eq_(CH_3_ and O).

### Crystal data

**Table:**

C_19_H_13_N_3_O·CH_4_O	*F*(000) = 348
*M**_r_* = 331.37	*D*_x_ = 1.365 Mg m^−^^3^
Monoclinic, *P**c*	Cu *K*α radiation, λ = 1.54184 Å
Hall symbol: P -2yc	Cell parameters from 1347 reflections
*a* = 11.9398 (12) Å	θ = 3.1–75.2°
*b* = 4.6680 (5) Å	µ = 0.73 mm^−^^1^
*c* = 14.7818 (18) Å	*T* = 123 K
β = 101.961 (11)°	Triangular plate, yellow
*V* = 805.98 (16) Å^3^	1.15 × 0.84 × 0.06 mm
*Z* = 2	

### Data collection

**Table:**

Oxford Diffraction Gemini-R diffractometer	1960 independent reflections
Radiation source: Enhance (Cu) X-ray Source	1885 reflections with *I* > 2σ(*I*)
Graphite monochromator	*R*_int_ = 0.030
Detector resolution: 10.5081 pixels mm^-1^	θ_max_ = 75.2°, θ_min_ = 3.1°
ω scans	*h* = −13→14
Absorption correction: analytical [*CrysAlis RED* (Oxford Diffraction, 2007), using a multi-faceted crystal model (Clark & Reid, 1995)]	*k* = −5→5
*T*_min_ = 0.505, *T*_max_ = 0.954	*l* = −18→12
3176 measured reflections	

### Refinement

**Table:**

Refinement on *F*^2^	Secondary atom site location: difference Fourier map
Least-squares matrix: full	Hydrogen site location: inferred from neighbouring sites
*R*[*F*^2^ > 2σ(*F*^2^)] = 0.039	H-atom parameters constrained
*wR*(*F*^2^) = 0.110	*w* = 1/[σ^2^(*F*_o_^2^) + (0.0747*P*)^2^ + 0.0897*P*] where *P* = (*F*_o_^2^ + 2*F*_c_^2^)/3
*S* = 1.04	(Δ/σ)_max_ < 0.001
1960 reflections	Δρ_max_ = 0.24 e Å^−^^3^
229 parameters	Δρ_min_ = −0.17 e Å^−^^3^
2 restraints	Absolute structure: Flack, H. D. (1983). *Acta Cryst.* A39, 876–881, 303 Friedel pairs
Primary atom site location: structure-invariant direct methods	Flack parameter: −1.5 (18)

### Special details

**Table:**

Experimental. The crystal was very fragile. On cutting the crystal shattered so an incident collimator of 1.0 mm was used. IR (KBr): 3435(Ar—OH), 3020(Ar), 1616 (C=N—C). ^13^ C NMR, 167 (C—OH), 165 (C=C—N), 150,152 and 148 (C=N) p.p.m.. LC—MS, m/*z* (%): 298 (*M*-1). In the electronic spectrum two band appears at 281 and 340 nm which can be assigned to the π -π * and n-π * transition of C=C and C=N group.The FTIR spectra were obtained on a Perkin Elmer Spectrum One Bv 5.0 spectrophotometer. ^1^H NMR and ^13^C NMR spectra were recorded on a Varian UNITY INOVA 500 MHz s pectrometer. Mass spectra were measured on a FinniganTM LCQTM Advantage MAX spectrometer. Electronic spectra were obtained on a Agilent 8453 UV-Vis. Spectroscopy System. Elemental analyses were obtained on a Thermo Finnigan Flash EA 112. All other chemicals employed were of the highest grade available.Absorption correction: CrysAlis RED, (Oxford Diffraction, 2007) Analytical numeric absorption correction using a multifaceted crystal model (Clark & Reid, 1995).
Geometry. All e.s.d.'s (except the e.s.d. in the dihedral angle between two l.s. planes) are estimated using the full covariance matrix. The cell e.s.d.'s are taken into account individually in the estimation of e.s.d.'s in distances, angles and torsion angles; correlations between e.s.d.'s in cell parameters are only used when they are defined by crystal symmetry. An approximate (isotropic) treatment of cell e.s.d.'s is used for estimating e.s.d.'s involving l.s. planes.
Refinement. Refinement of *F*^2^ against ALL reflections. The weighted *R*-factor *wR* and goodness of fit *S* are based on *F*^2^, conventional *R*-factors *R* are based on *F*, with *F* set to zero for negative *F*^2^. The threshold expression of *F*^2^ > σ(*F*^2^) is used only for calculating *R*-factors(gt) *etc*. and is not relevant to the choice of reflections for refinement. *R*-factors based on *F*^2^ are statistically about twice as large as those based on *F*, and *R*- factors based on ALL data will be even larger.

### Fractional atomic coordinates and isotropic or equivalent isotropic displacement parameters (Å^2^)

**Table:**

	*x*	*y*	*z*	*U*_iso_*/*U*_eq_	
O1	0.98484 (16)	1.4695 (5)	0.63127 (14)	0.0337 (4)	
H1	1.0514	1.5260	0.6545	0.051*	
O1S	1.18635 (15)	1.6610 (4)	0.71862 (14)	0.0307 (4)	
H1S	1.2497	1.6321	0.7038	0.046*	
N1	0.41348 (18)	0.5215 (5)	0.70687 (15)	0.0254 (4)	
N2	0.32594 (18)	0.8980 (5)	0.56783 (15)	0.0268 (4)	
N3	0.70428 (17)	1.0456 (5)	0.50248 (15)	0.0256 (4)	
C1	0.4567 (2)	0.3337 (6)	0.77219 (17)	0.0275 (5)	
H1A	0.4061	0.2468	0.8058	0.033*	
C2	0.5729 (2)	0.2556 (6)	0.79469 (18)	0.0291 (5)	
H2A	0.5997	0.1206	0.8424	0.035*	
C3	0.6463 (2)	0.3780 (5)	0.74647 (18)	0.0265 (5)	
H3A	0.7252	0.3282	0.7600	0.032*	
C4	0.60430 (19)	0.5796 (5)	0.67619 (16)	0.0233 (5)	
C5	0.6784 (2)	0.7166 (5)	0.62454 (17)	0.0237 (5)	
H5A	0.7577	0.6711	0.6372	0.028*	
C6	0.63612 (19)	0.9123 (5)	0.55719 (16)	0.0234 (5)	
C7	0.5151 (2)	0.9788 (5)	0.53588 (16)	0.0227 (5)	
C8	0.4682 (2)	1.1790 (6)	0.46665 (17)	0.0258 (5)	
H8A	0.5159	1.2761	0.4326	0.031*	
C9	0.3527 (2)	1.2309 (6)	0.44944 (19)	0.0299 (5)	
H9A	0.3191	1.3639	0.4030	0.036*	
C10	0.2852 (2)	1.0854 (6)	0.50113 (19)	0.0297 (5)	
H10A	0.2052	1.1223	0.4879	0.036*	
C11	0.4404 (2)	0.8465 (5)	0.58528 (16)	0.0233 (5)	
C12	0.48604 (19)	0.6424 (5)	0.65842 (16)	0.0221 (5)	
C13	0.8014 (2)	1.1466 (6)	0.54239 (17)	0.0252 (5)	
H13A	0.8229	1.1375	0.6079	0.030*	
C14	0.8805 (2)	1.2756 (5)	0.49080 (18)	0.0258 (5)	
C15	0.9738 (2)	1.4372 (5)	0.53890 (18)	0.0268 (5)	
C16	1.0503 (2)	1.5581 (6)	0.4904 (2)	0.0311 (5)	
H16A	1.1138	1.6655	0.5226	0.037*	
C17	1.0351 (2)	1.5235 (6)	0.3963 (2)	0.0358 (6)	
H17A	1.0878	1.6080	0.3641	0.043*	
C18	0.9425 (2)	1.3650 (8)	0.3476 (2)	0.0382 (6)	
H18A	0.9323	1.3405	0.2826	0.046*	
C19	0.8661 (2)	1.2446 (6)	0.39525 (18)	0.0305 (5)	
H19A	0.8027	1.1387	0.3623	0.037*	
C1S	1.1695 (2)	1.9603 (6)	0.7274 (2)	0.0368 (6)	
H1S1	1.2045	2.0217	0.7903	0.055*	
H1S2	1.0873	2.0020	0.7150	0.055*	
H1S3	1.2051	2.0636	0.6829	0.055*	

### Atomic displacement parameters (Å^2^)

**Table:**

	*U*^11^	*U*^22^	*U*^33^	*U*^12^	*U*^13^	*U*^23^
O1	0.0270 (8)	0.0449 (11)	0.0295 (9)	−0.0100 (8)	0.0067 (7)	−0.0060 (8)
O1S	0.0226 (8)	0.0333 (9)	0.0364 (10)	−0.0025 (7)	0.0064 (7)	−0.0027 (8)
N1	0.0220 (9)	0.0255 (10)	0.0282 (10)	−0.0003 (8)	0.0040 (8)	−0.0010 (9)
N2	0.0228 (9)	0.0262 (10)	0.0310 (11)	0.0006 (8)	0.0047 (8)	0.0015 (8)
N3	0.0223 (10)	0.0280 (10)	0.0269 (10)	−0.0002 (8)	0.0061 (8)	−0.0011 (8)
C1	0.0298 (12)	0.0264 (12)	0.0271 (12)	−0.0032 (10)	0.0081 (10)	0.0005 (10)
C2	0.0306 (12)	0.0284 (12)	0.0262 (12)	−0.0009 (10)	0.0011 (9)	0.0011 (9)
C3	0.0243 (11)	0.0253 (11)	0.0277 (12)	0.0021 (9)	0.0002 (9)	−0.0014 (10)
C4	0.0227 (12)	0.0209 (10)	0.0255 (12)	−0.0011 (9)	0.0029 (9)	−0.0024 (9)
C5	0.0203 (10)	0.0243 (11)	0.0260 (11)	0.0004 (9)	0.0035 (8)	−0.0032 (9)
C6	0.0236 (11)	0.0220 (10)	0.0244 (11)	−0.0013 (9)	0.0044 (9)	−0.0041 (9)
C7	0.0227 (10)	0.0212 (10)	0.0233 (11)	−0.0013 (8)	0.0025 (9)	−0.0029 (9)
C8	0.0286 (12)	0.0246 (11)	0.0242 (11)	−0.0008 (9)	0.0053 (9)	−0.0010 (9)
C9	0.0309 (13)	0.0298 (11)	0.0272 (11)	0.0032 (10)	0.0022 (10)	0.0041 (10)
C10	0.0225 (12)	0.0290 (12)	0.0363 (13)	0.0035 (9)	0.0032 (10)	0.0019 (11)
C11	0.0221 (11)	0.0217 (10)	0.0250 (11)	−0.0013 (8)	0.0024 (9)	−0.0033 (9)
C12	0.0206 (10)	0.0206 (11)	0.0246 (11)	−0.0011 (8)	0.0034 (9)	−0.0023 (9)
C13	0.0241 (11)	0.0259 (11)	0.0257 (11)	0.0017 (9)	0.0057 (8)	0.0000 (9)
C14	0.0214 (10)	0.0268 (11)	0.0294 (12)	0.0020 (9)	0.0056 (9)	0.0022 (10)
C15	0.0222 (10)	0.0275 (11)	0.0308 (12)	0.0021 (9)	0.0056 (9)	0.0002 (10)
C16	0.0215 (11)	0.0326 (13)	0.0397 (14)	−0.0031 (10)	0.0072 (10)	0.0021 (11)
C17	0.0248 (11)	0.0432 (14)	0.0417 (15)	0.0030 (11)	0.0123 (10)	0.0150 (12)
C18	0.0313 (13)	0.0560 (18)	0.0273 (12)	0.0040 (12)	0.0061 (10)	0.0063 (12)
C19	0.0228 (11)	0.0403 (14)	0.0279 (12)	−0.0011 (10)	0.0039 (9)	0.0007 (11)
C1S	0.0318 (13)	0.0350 (14)	0.0451 (16)	−0.0016 (11)	0.0115 (11)	−0.0054 (12)

### Geometric parameters (Å, º)

**Table:**

O1—C15	1.353 (3)	C7—C8	1.413 (3)
O1—H1	0.8400	C8—C9	1.371 (4)
O1S—C1S	1.421 (3)	C8—H8A	0.9500
O1S—H1S	0.8400	C9—C10	1.396 (4)
N1—C1	1.327 (3)	C9—H9A	0.9500
N1—C12	1.356 (3)	C10—H10A	0.9500
N2—C10	1.333 (3)	C11—C12	1.459 (3)
N2—C11	1.359 (3)	C13—C14	1.462 (3)
N3—C13	1.277 (3)	C13—H13A	0.9500
N3—C6	1.406 (3)	C14—C19	1.395 (4)
C1—C2	1.406 (4)	C14—C15	1.410 (3)
C1—H1A	0.9500	C15—C16	1.393 (3)
C2—C3	1.365 (4)	C16—C17	1.374 (4)
C2—H2A	0.9500	C16—H16A	0.9500
C3—C4	1.414 (4)	C17—C18	1.399 (4)
C3—H3A	0.9500	C17—H17A	0.9500
C4—C12	1.412 (3)	C18—C19	1.382 (4)
C4—C5	1.434 (3)	C18—H18A	0.9500
C5—C6	1.368 (3)	C19—H19A	0.9500
C5—H5A	0.9500	C1S—H1S1	0.9800
C6—C7	1.447 (3)	C1S—H1S2	0.9800
C7—C11	1.407 (3)	C1S—H1S3	0.9800
			
C15—O1—H1	109.5	C9—C10—H10A	118.0
C1S—O1S—H1S	109.5	N2—C11—C7	123.0 (2)
C1—N1—C12	117.7 (2)	N2—C11—C12	117.5 (2)
C10—N2—C11	117.1 (2)	C7—C11—C12	119.5 (2)
C13—N3—C6	118.4 (2)	N1—C12—C4	122.6 (2)
N1—C1—C2	124.0 (2)	N1—C12—C11	118.80 (19)
N1—C1—H1A	118.0	C4—C12—C11	118.6 (2)
C2—C1—H1A	118.0	N3—C13—C14	122.3 (2)
C3—C2—C1	118.5 (2)	N3—C13—H13A	118.9
C3—C2—H2A	120.8	C14—C13—H13A	118.9
C1—C2—H2A	120.8	C19—C14—C15	119.0 (2)
C2—C3—C4	119.6 (2)	C19—C14—C13	121.9 (2)
C2—C3—H3A	120.2	C15—C14—C13	119.1 (2)
C4—C3—H3A	120.2	O1—C15—C16	122.5 (2)
C12—C4—C3	117.6 (2)	O1—C15—C14	118.0 (2)
C12—C4—C5	120.8 (2)	C16—C15—C14	119.4 (2)
C3—C4—C5	121.6 (2)	C17—C16—C15	120.7 (2)
C6—C5—C4	120.6 (2)	C17—C16—H16A	119.7
C6—C5—H5A	119.7	C15—C16—H16A	119.7
C4—C5—H5A	119.7	C16—C17—C18	120.5 (3)
C5—C6—N3	122.9 (2)	C16—C17—H17A	119.7
C5—C6—C7	120.2 (2)	C18—C17—H17A	119.7
N3—C6—C7	116.8 (2)	C19—C18—C17	119.2 (3)
C11—C7—C8	117.9 (2)	C19—C18—H18A	120.4
C11—C7—C6	120.3 (2)	C17—C18—H18A	120.4
C8—C7—C6	121.8 (2)	C18—C19—C14	121.2 (2)
C9—C8—C7	118.9 (2)	C18—C19—H19A	119.4
C9—C8—H8A	120.5	C14—C19—H19A	119.4
C7—C8—H8A	120.5	O1S—C1S—H1S1	109.5
C8—C9—C10	119.0 (2)	O1S—C1S—H1S2	109.5
C8—C9—H9A	120.5	H1S1—C1S—H1S2	109.5
C10—C9—H9A	120.5	O1S—C1S—H1S3	109.5
N2—C10—C9	124.1 (2)	H1S1—C1S—H1S3	109.5
N2—C10—H10A	118.0	H1S2—C1S—H1S3	109.5
			
C12—N1—C1—C2	0.7 (4)	C6—C7—C11—C12	0.2 (3)
N1—C1—C2—C3	−0.4 (4)	C1—N1—C12—C4	−1.2 (3)
C1—C2—C3—C4	0.4 (4)	C1—N1—C12—C11	179.1 (2)
C2—C3—C4—C12	−0.8 (3)	C3—C4—C12—N1	1.2 (3)
C2—C3—C4—C5	179.2 (2)	C5—C4—C12—N1	−178.7 (2)
C12—C4—C5—C6	0.3 (3)	C3—C4—C12—C11	−179.1 (2)
C3—C4—C5—C6	−179.6 (2)	C5—C4—C12—C11	1.0 (3)
C4—C5—C6—N3	−177.8 (2)	N2—C11—C12—N1	−2.0 (3)
C4—C5—C6—C7	−1.3 (3)	C7—C11—C12—N1	178.5 (2)
C13—N3—C6—C5	−46.2 (3)	N2—C11—C12—C4	178.3 (2)
C13—N3—C6—C7	137.3 (2)	C7—C11—C12—C4	−1.2 (3)
C5—C6—C7—C11	1.1 (3)	C6—N3—C13—C14	176.8 (2)
N3—C6—C7—C11	177.8 (2)	N3—C13—C14—C19	−13.4 (4)
C5—C6—C7—C8	−179.8 (2)	N3—C13—C14—C15	166.3 (2)
N3—C6—C7—C8	−3.1 (3)	C19—C14—C15—O1	178.7 (2)
C11—C7—C8—C9	−1.3 (3)	C13—C14—C15—O1	−1.0 (3)
C6—C7—C8—C9	179.6 (2)	C19—C14—C15—C16	−1.0 (3)
C7—C8—C9—C10	0.3 (4)	C13—C14—C15—C16	179.3 (2)
C11—N2—C10—C9	−0.4 (4)	O1—C15—C16—C17	−179.1 (2)
C8—C9—C10—N2	0.6 (4)	C14—C15—C16—C17	0.7 (4)
C10—N2—C11—C7	−0.7 (3)	C15—C16—C17—C18	−0.3 (4)
C10—N2—C11—C12	179.8 (2)	C16—C17—C18—C19	0.3 (4)
C8—C7—C11—N2	1.6 (3)	C17—C18—C19—C14	−0.7 (4)
C6—C7—C11—N2	−179.3 (2)	C15—C14—C19—C18	1.1 (4)
C8—C7—C11—C12	−178.9 (2)	C13—C14—C19—C18	−179.2 (3)

### Hydrogen-bond geometry (Å, º)

**Table:**

*D*—H···*A*	*D*—H	H···*A*	*D*···*A*	*D*—H···*A*
O1—H1···O1*S*	0.84	1.81	2.640 (3)	172
O1*S*—H1*S*···N1^i^	0.84	2.01	2.829 (3)	163
O1*S*—H1*S*···N2^i^	0.84	2.68	3.242 (3)	126

Symmetry code: (i) *x*+1, *y*+1, *z*.

## References

[bb1] Allen, F. H., Kennard, O., Watson, D. G., Brammer, L., Orpen, A. G. & Taylor, R. (1987). *J. Chem. Soc. Perkin Trans. 2*, pp. S1–19.

[bb2] Avciata, U., Bozdogan, A. E., Kocak, M., Gul, A. & Bekaroglu, O. (1995). *J. Coord. Chem.* **35**, 319–323.

[bb3] Avciata, U., Demirhan, N. & Gül, A. (1998). *Monatsh. Chem.* **29**, 9–18.

[bb4] Balzani, V., Juris, A., Campagna, S. & Serroni, S. (1996). *Chem. Rev.* pp. 759–833. 10.1021/cr941154y 11848772

[bb5] Bolger, J., Gourdon, A., Ishow, E. & Launay, J. P. (1996). *Inorg. Chem.* **35**, 2937–2944.

[bb6] Camren, H., Chang, M. Y., Zeng, L. & Mc Guire, M. E. (1996). *Synth. Commun.* **26**, 1247–1252.

[bb7] Can, S. & Bekaroglu, O. (1988). *J. Chem. Soc. Dalton Trans.* pp. 2831–2835.

[bb8] Clark, R. C. & Reid, J. S. (1995). *Acta Cryst.* A**51**, 887–897.

[bb9] Demirhan, N., Erden, I. & Avciata, U. (2002). *Synth. React. Inorg. Met. Org. Chem.* **32**, 1567–1577.

[bb11] Fun, H.-K., Loh, W.-S., Maity, A. C. & Goswami, S. (2010). *Acta Cryst.* E**66**, o1320. 10.1107/S1600536810016405 PMC2979584 21579413

[bb12] Gillard, R. D. & Hill, R. E. E. (1974). *J. Chem. Soc. Dalton Trans.* pp. 1217–1236.

[bb13] Gul, A., Okur, A. I., Cihan, A., Tan, N. & Bekaroglu, O. (1986). *Synth. React. Inorg. Met. Org. Chem.* **16**, 871–884.

[bb14] Hobday, M. D. & Smith, T. S. (1973). *Coord. Chem. Rev.* **9**, 311–337.

[bb15] Msood, A. & Hodgson, D. J. (1993). *Inorg. Chem.* **32**, 4839–4844.

[bb16] Oxford Diffraction (2007). *CrysAlis PRO* and *CrysAlis RED* Oxford Diffraction Ltd, Abingdon, England.

[bb17] Sammes, P. G. & Yahioglu, G. (1994). *Chem. Soc. Rev.* **23**, 327–336.

[bb18] Sheldrick, G. M. (2008). *Acta Cryst.* A**64**, 112–122. 10.1107/S0108767307043930 18156677

[bb19] Wu, X.-Y., Xu, X.-J. & Wang, X.-C. (2011). *Acta Cryst.* E**67**, o474. 10.1107/S1600536811002492 PMC3051702 21523132

